# Identification of Catalposide Metabolites in Human Liver and Intestinal Preparations and Characterization of the Relevant Sulfotransferase, UDP-glucuronosyltransferase, and Carboxylesterase Enzymes

**DOI:** 10.3390/pharmaceutics11070355

**Published:** 2019-07-22

**Authors:** Deok-Kyu Hwang, Ju-Hyun Kim, Yongho Shin, Won-Gu Choi, Sunjoo Kim, Yong-Yeon Cho, Joo Young Lee, Han Chang Kang, Hye Suk Lee

**Affiliations:** 1 BK21 PLUS Team for Creative Leader Program for Pharmacomics-based Future Pharmacy, College of Pharmacy, The Catholic University of Korea, Bucheon 14662, Korea; 2 College of Pharmacy, Yeungnam University, Gyeongsan 38541, Korea

**Keywords:** catalposide, in vitro human metabolism, UDP-glucuronosyltransferase, sulfotransferase, carboxylesterase

## Abstract

Catalposide, an active component of *Veronica* species such as *Catalpa ovata* and *Pseudolysimachion lingifolium*, exhibits anti-inflammatory, antinociceptic, anti-oxidant, hepatoprotective, and cytostatic activities. We characterized the in vitro metabolic pathways of catalposide to predict its pharmacokinetics. Catalposide was metabolized to catalposide sulfate (M1), 4-hydroxybenzoic acid (M2), 4-hydroxybenzoic acid glucuronide (M3), and catalposide glucuronide (M4) by human hepatocytes, liver S9 fractions, and intestinal microsomes. M1 formation from catalposide was catalyzed by sulfotransferases (SULTs) 1C4, SULT1A1*1, SULT1A1*2, and SULT1E1. Catalposide glucuronidation to M4 was catalyzed by gastrointestine-specific UDP-glucuronosyltransferases (UGTs) 1A8 and UGT1A10; M4 was not detected after incubation of catalposide with human liver preparations. Hydrolysis of catalposide to M2 was catalyzed by carboxylesterases (CESs) 1 and 2, and M2 was further metabolized to M3 by UGT1A6 and UGT1A9 enzymes. Catalposide was also metabolized in extrahepatic tissues; genetic polymorphisms of the carboxylesterase (CES), UDP-glucuronosyltransferase (UGT), and sulfotransferase (SULT) enzymes responsible for catalposide metabolism may cause inter-individual variability in terms of catalposide pharmacokinetics.

## 1. Introduction

Catalposide is an active iridoid glycoside of *Veronica* species including *Catalpa ovata* and *Pseudolysimachion lingifolium* [1,2,3]. Catalposide exhibits various biological effects including anti-inflammatory [4,5,6,7,8,9], anti-oxidant [10], antinociceptic [8], cytostatic [11], hypolipidemic via peroxisome proliferator-activated receptor-α activation [12], and hepatoprotective activities [13].

Catalposide had a short half-life (19.3 ± 9.5 min), and exhibited high systemic clearance (96.7 ± 44.1 mL/min/kg), and low urinary excretion (9.9 ± 4.1% of the dose) after intravenous administration of 10 mg/kg to male Sprague-Dawley rats [14]. This indicated that catalposide might be extensively metabolized in rats. However, catalposide remained stable after 1 h incubation with rat liver microsomes in the presence of NADPH [14]. Thus, catalposide may be catabolized via non-cytochrome P450 (CYP)-mediated mechanism. Catalposide was the substrate of OAT3, OATP1B1, and OATP1B3 transporters and weakly inhibited their transport activities with IC_50_ values of 83, 200, and 235 μM, respectively, suggesting that OAT3, OATP1B1, and OATP1B3 may regulate the pharmacokinetics and drug interactions of catalposide [15].

Pharmacokinetics and metabolism of potential active constituents in herbal drugs is helpful for the determination of dosage regimens and interpretation of pharmacological effects under clinical conditions [16]. It is important to establish the comparative metabolism and drug-metabolizing enzymes of active constituents for a full characterization of its pharmacokinetics, pharmacodynamics, and toxicity. The characterization of drug-metabolizing enzymes such as CYPs, carboxylesterases (CESs), UDP-glucuronosyltransferases (UGTs), and sulfotransferases (SULTs) responsible for the metabolism of a drug may reveal inter-individual variability in drug metabolism and the potential drug interactions [17,18,19]. However, catalposide metabolism has not been studied in humans and animals. We identified catalposide metabolites and drug-metabolizing enzymes involved to predict its pharmacokinetics and possible drug interactions.

We identified catalposide metabolites formed from in vitro incubations of catalposide with human hepatocytes, intestinal microsomes, and liver S9 fractions using liquid chromatography-high resolution mass spectrometry (LC-HRMS) and characterized the CES, UGT, and SULT enzymes involved in catalposide metabolism using human cDNA-expressed CES, UGT, and SULT supersomes, respectively. 

## 2. Materials and Methods 

### 2.1. Materials and Reagents

Catalposide (purity, 98%) was obtained from Aobious Inc. (Gloucester, MA, USA). Alamethicin, 3-phosphoadenosine-5-phosphosulfate (PAPS), and uridine 5′-diphosphoglucuronic acid (UDPGA) were from Sigma-Aldrich Co. (St. Louis, MO, USA). 4-hydroxybenzoic acid and 4-hydroxybenzoic acid glucuronide were purchased from Toronto Research Chemicals (North York, ON, Canada). Pooled human intestinal microsomes; pooled human liver S9 fractions; human cDNA-expressed UGTs 1A1/3/4/6/7/8/9/10 and 2B4/7/15/17 supersomes; human cDNA-expressed CESs 1b, 1c, and 2 supersomes; cryopreserved human hepatocytes; and hepatocyte purification kits were obtained from Corning Life Sciences (Woburn, MA, USA). Human cDNA-expressed SULT 1A1*1, 1A1*2, 1A2, 1A3, 1B1, 1C2, 1C4, 1E1, and 2A1 supersomes were purchased from Cypex Ltd. (Dundee, UK). Methanol (HPLC grade) was from Burdick & Jackson Inc. (SK Chemicals, Ulsan, Korea), and all other chemicals were of the highest quality available. Calibration mixtures for Exactive MS [ProteoMass LTQ/FT-hybrid ESI positive mode Cal Mix (MSCAL5) and negative mode Cal Mix (MSCAL6)] were obtained from Supelco (Bellefonte, PA, USA). 

### 2.2. In Vitro Metabolism of Catalposide in Cryopreserved Human Hepatocytes

Cryopreserved human hepatocytes were recovered with the aid of a hepatocyte purification kit, and viable cells were resuspended in William’s E buffer at a final concentration of 1.28 × 10^6^ cells/mL [20]. Human hepatocyte suspensions (62.5 µL, 8.00 × 10^4^ cells) and 62.5 µL of 400 µM catalposide in William’s E buffer were added to the wells of a 96-well plate and the mixture was incubated for 120 min at 37 °C in a CO_2_ incubator. Methanol (250 µL) was added to each well and the mixture was centrifuged at 3000× *g* for 10 min. Aliquots of the supernatants (250 µL) were evaporated to dryness using a vacuum evaporator (Genevac Ltd., Ipswich, UK). Each residue was dissolved in 100 µL of 5% methanol and an aliquot (5 µL) was injected into the LC-HRMS system.

### 2.3. In Vitro Metabolism of Catalposide in Human Liver S9 Fractions and Intestinal Microsomes

Each reaction mixture contained 50 mM potassium phosphate buffer (pH 7.4), 10 mM magnesium chloride, human liver S9 fractions or human intestinal microsomes (100 μg protein), 2 mM UDPGA or 200 μM PAPS, 200 μM catalposide or a possible metabolite, and 1000 μM 4-hydroxybenzoic acid in a volume of 200 μL. Samples lacking UDPGA and PAPS served as controls. The mixtures were incubated at 37 °C for 60 min and the reactions were then quenched by adding 500 μL of methanol. The tubes were centrifuged and the supernatants evaporated to dryness using a vacuum concentrator. The residues were dissolved in 100 μL of 5% methanol and 5 μL aliquots were injected into the LC-HRMS system. 

### 2.4. Characterization of Human SULTs Involved in Catalposide Sulfation

To screen for SULT enzymes involved in catalposide sulfation, each 100 μL of reaction mixture contained 20 μM PAPS; 10 mM dithiothreitol; 5 mM magnesium chloride; 50 mM phosphate buffer (pH 7.4); 150 μM catalposide; and the human liver S9 fraction (40 μg protein) or human cDNA-expressed SULT 1A1*1, 1A1*2, 1A2, 1A3, 2A1, 1B1, 1C2, 1C4, or 1E1 supersomes [1A1*1 (0.25), 1A1*2 (0.5), 1A2 (0.25), 1A3 (0.2), 2A1 (1.25), 1B1 (0.5), 1C2 (2.5), 1C4 (0.1), 1E1 (0.2 μg protein)]. Incubation proceeded at 37 °C for 5 min. The reactions were stopped by adding 100 μL of methanol containing 30 ng/mL 4-methylumbelliferone (an internal standard). After vortex-mixing and centrifugation, 50 μL of each supernatant was diluted with 50 μL deionized water. The mixture was transferred to an injection vial, and an aliquot (5 μL) was injected into the LC-HRMS system. 

To explore the kinetics of the metabolism of catalposide to catalposide sulfate, various concentrations of catalposide (10 to 2000 μM) were incubated in duplicate with pooled human liver S9 fractions (40 μg protein) or human cDNA-expressed SULT1A1*1 (0.5 μg protein), SULT1A1*2 (0.5 μg protein), SULT1C4 (0.1 μg protein), or SULT1E1 (0.2 μg protein) in the presence of 20 μM PAPS, 10 mM dithiothreitol, and 5 mM magnesium chloride at 37 °C for 5 min to obtain *K*_m_ and *V*_max_ values.

### 2.5. Characterization of Human UGTs Involved in Catalposide and 4-Hydroxybenzoic Acid Glucuronidation

To identify the UGT enzymes responsible for formation of catalposide glucuronide (M4) from catalposide and 4-hydroxybenzoic acid glucuronide (M3) from 4-hydroxybenzoic acid (M2), 100 μL reaction mixtures containing human intestinal microsomes (40 μg protein), or human cDNA-expressed UGTs 1A1, 1A3, 1A4, 1A6, 1A7, 1A8, 1A9, 1A10, 2B4, 2B7, 2B15, or 2B17 (10 μg protein); 2 mM UDPGA; 0.025 mg/mL alamethicin; and 400 μM catalposide or 500 μM 4-hydroxybenzoic acid in 50 mM Tris buffer (pH 7.4) were incubated at 37 °C for 60 min. The reactions were stopped by addition of 100 μL methanol containing 30 ng/mL 4-methylumbelliferone (an internal standard). After vortex-mixing and centrifugation, 50 μL of each supernatant was diluted with an equal volume of water and a 5 μL aliquot was injected into the LC-HRMS system.

To explore the kinetics of the metabolism of catalposide to catalposide glucuronide, various concentrations of catalposide (10, 25, 100, 400, 800, 1200, 1600, and 2000 μM) were incubated in duplicate with 2 mM UDPGA, 0.025 mg/mL alamethicin, pooled human intestinal microsomes (40 μg protein), or human cDNA-expressed UGT1A8 or UGT1A10 supersomes (20 μg protein), to obtain *K*_m_ and *V*_max_ values.

### 2.6. Characterization of Carboxylesterases Involved in the Formation of 4-Hydroxybenzoic Acid from Catalposide 

To identify the CES enzymes involved in hydrolysis of catalposide to 4-hydroxybenzoic acid, 100 μL reaction mixtures containing human liver S9 fractions; human intestinal microsomes; or human CES1b, CES1c, or CES2 enzymes (50 μg protein), and catalposide (200 or 400 μM) in 50 mM phosphate buffer (pH 7.4) were incubated at 37 °C for 30 min. Reactions were stopped by addition of 100 μL 4-methylumbelliferone (internal standard, 10 ng/mL) in methanol. After vortex-mixing and centrifugation, 50 μL of each supernatant was diluted with 50 μL of deionized water. Each mixture was transferred to an injection vial, and a 5 μL aliquot was injected into the LC-HRMS system. 

### 2.7. LC-HRMS Analysis of Catalposide and Metabolites 

To separate and identify catalposide and its metabolites, we used a Q-Exactive Orbitrap mass spectrometer coupled to an Accela UPLC system (Thermo Scientific, Waltham, MA, USA). Catalposide and its metabolites were optimally separated on a Halo C18 column via gradient elution using 5% (*v*/*v*) methanol in 1 mM ammonium formate (pH 3.1) (mobile phase A) and methanol (mobile phase B) at a flow rate of 0.5 mL/min: 5% mobile phase B for 2 min, 5–20% mobile phase B over 11.5 min, 20–90% mobile phase B over 0.5 min, 90% mobile phase B for 3 min, 90–5% mobile phase B over 0.5 min, and 5% mobile phase B for 2.5 min. The column and the autosampler were maintained at 40 and 6 °C, respectively. Accurate mass measurements of catalposide and its metabolites were derived via electrospray ionization in the negative mode using the following electrospray source settings: ion transfer capillary temperature, 330 °C; needle spray voltage, −3000 V; capillary voltage, −47.5 V; nitrogen sheath gas, 50 arbitrary units; auxiliary gas, 15 arbitrary units. The resolution and automatic gain control were scaled to 70,000 and 1,000,000, respectively. MS data were obtained using external calibration over the scan range *m/z* 100–700 and processed using Xcalibur software version 2.2 (Thermo Scientific). The Q-Exactive Orbitrap MS was calibrated using MSCAL5 and MSCAL6 for the positive and negative ion modes, respectively. Nitrogen gas was employed for higher-energy collision dissociation (HCD) at an energy of 25 eV to obtain product ion spectra of catalposide and its metabolites. Structures were determined using Mass Frontier software (version 6.0; HighChem Ltd., Bratislava, Slovakia). We used the extracted ion monitoring mode for quantification: *m/z* 481.1349 for catalposide, *m/z* 657.1674 for catalposide glucuronide, *m/z* 561.0921 for catalposide sulfate, *m/z* 137.0239 for 4-hydroxybenzoic acid, *m/z* 313.0569 for 4-hydroxybenzoic acid glucuronide, and *m/z* 175.0410 for 4-methylumbelliferone (the internal standard). The peak areas of all components were integrated using Xcalibur software. The calibration curve was linear over the catalposide concentration range 0.5–200 pmol. The concentrations of catalposide glucuronide and catalposide sulfate were calculated using the calibration curve for catalposide because we had no authentic standards.

### 2.8. Data Analysis 

All results are the average of two determinations obtained using pooled human intestinal microsomes, pooled human liver S9 fractions, UGTs, and SULTs. The apparent kinetic parameters (*K*_m_, *V*_max_, n, and *K*_i_) for formation of catalposide glucuronide or catalposide sulfate by human intestinal microsomes, liver S9 fractions, UGTs, or SULTs were determined by fitting the Hill equation model [*V* = *V*_max_S^n^/(*K*_m_^n^ + S^n^)], the substrate inhibition model [*V* = *V*_max_/(1 + *K*_m_/S + S/*K*_i_)], or the single enzyme model [*V* = *V*_max_S/(*K*_m_ + S)] to the unweighted formation rates of catalposide glucuronide and catalposide sulfate, respectively, over a range of catalposide concentrations using Enzyme Kinetics software (version 1.1 SPSS Science Inc., Chicago, IL, USA). In the above equations, *V* is the velocity of the reaction at substrate concentration [S], *V*_max_ is the maximum velocity, n is the Hill constant, *K*_m_ is the substrate concentration at which the reaction velocity is 50% of *V*_max_, and *K*_i_ is the dissociation constant of the substrate binding to the inhibitory region within the enzyme active site.

## 3. Results

### 3.1. In Vitro Metabolic Profiles of Catalposide Incubated with Human Hepatocytes and Intestinal Microsomes

**Figure 1:**
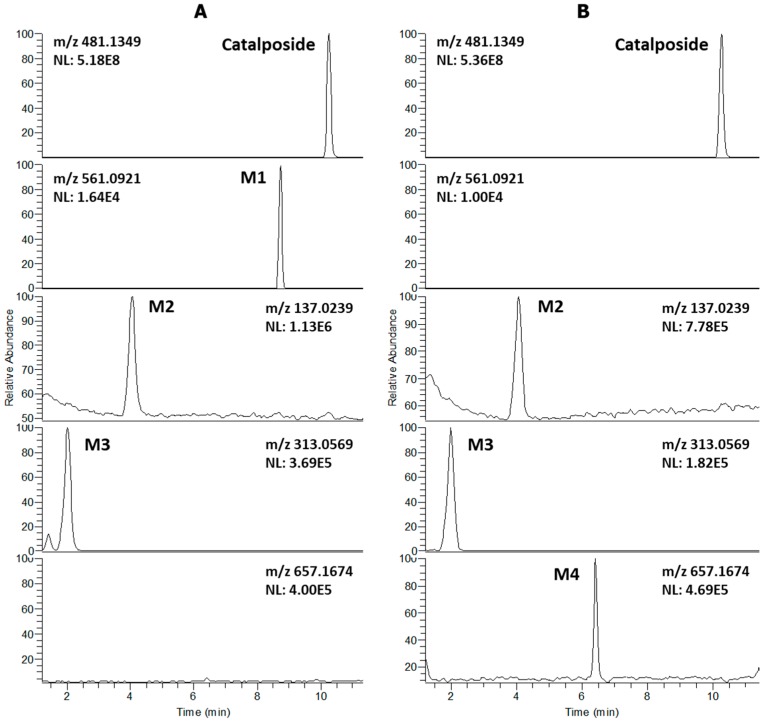
Extracted ion chromatograms of catalposide and its possible metabolites after incubation of 200 μM catalposide with (**A**) human hepatocytes for 2 h at 37 °C in a CO_2_ incubator and (**B**) human intestinal microsomes in the presence of UDPGA at 37 °C for 1 h (mass accuracy: 5 ppm). The extracted ion chromatograms were reconstructed based on the [M–H]^−^ ions: *m/z* 481.1349 for catalposide, *m/z* 561.0921 for M1 (catalposide sulfate), *m/z* 137.0239 for M2 (4-hydroxybenzoic acid), *m/z* 313.0569 for M3 (4-hydroxybenzoic acid glucuronide), and *m/z* 657.1674 for M4 (catalposide glucuronide).

LC-HRMS analysis of extracts after incubation of catalposide with human hepatocytes revealed three metabolites (M1–M3) and residual catalposide (Figure 1A). LC-HRMS analysis of reaction mixtures after incubation of catalposide with human intestinal microsomes in the presence of UDPGA yielded M2, M3, and a new metabolite M4 (Figure 1B).

**Figure 2:**
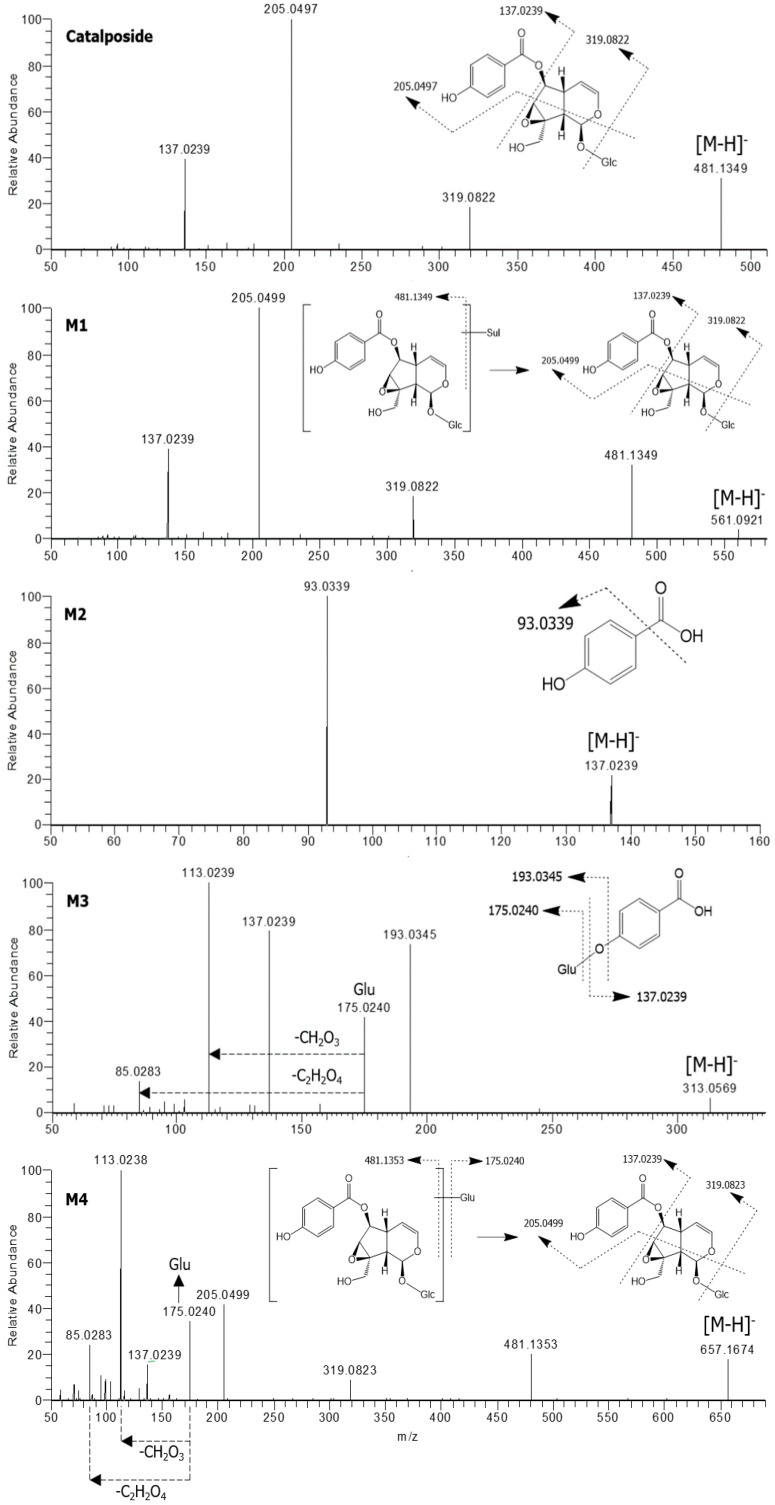
Product scan spectra of catalposide and its four metabolites, M1–M4 obtained via liquid chromatography-high resolution mass spectrometry (LC-HRMS) analysis of reaction mixtures obtained after incubation of catalposide with human intestinal microsomes in the presence of uridine 5′-diphosphoglucuronic acid (UDPGA) or human hepatocytes. Glc: glucose; Glu: glucuronosyl.

The formulae, deprotonated molecular ions ([M−H]^−^), mass errors, and retention times of catalposide and its four metabolites, M1–M4, are shown in Table 1. The four metabolite peaks were identified using the accurate mass values and the characteristic product ions of the product scan spectra (Table 1, Figure 2). The mass errors between the theoretical and observed *m/z* values for each metabolite were less than 5 ppm, indicating good correlations between the calculated theoretical masses and the experimentally observed masses obtained after full-scan MS analysis. 

The product scan spectra of catalposide exhibiting the [M−H]^−^ ion at *m/z* 481.1349 generated characteristic product ions at *m/z* 319.0822 (reflecting loss of glucose from the [M−H]^−^ ion), *m/z* 205.0497 (loss of C_5_H_6_O_3_ caused by the breakdown of the iridoid moiety of *m/z* 319.0822), and *m/z* 137.0239 (the 4-hydroxybenzoyl moiety) (Figure 2). 

M1 exhibited an [M−H]^−^ ion at *m/z* 561.0921, that is, 80 amu higher than the [M−H]^−^ ion of catalposide, indicating that M1 was catalposide sulfate. The product scan spectra of M1 generated the characteristic product ions at *m/z* 481.1349 (loss of SO_3_ from the [M−H]^−^ ion), *m/z* 319.0822 (loss of glucose from *m/z* 481.1349), *m/z* 205.0499, and *m/z* 137.0239 (Figure 2). M1 was also formed from catalposide after incubation with human liver S9 fractions in the presence of PAPS. Thus, M1 was identified as catalposide sulfate.

M2 exhibited an [M−H]^−^ ion at *m/z* 137.0239 and generated a characteristic product ion at *m/z* 93.0339 (reflecting loss of a carboxyl group from the [M−H]^−^ ion)(Figure 2). M2 was identified as 4-hydroxybenzoic acid by comparison with the mass, retention time, and product ion of the authentic standard.

M3 exhibited an [M−H]^−^ ion at *m/z* 313.0569, that is, 176 amu higher than the [M−H]^−^ ion of M2 (4-hydroxybenzoic acid), reflecting glucuronidation of M2. In the product scan spectrum of M3, characteristic product ions were observed at *m/z* 137.0239 (reflecting loss of the glucuronosyl moiety from the [M−H]^−^ ion), *m/z* 175.0240 (the glucuronosyl moiety), and *m/z* 113.0239 (loss of CO_2_ and H_2_O from *m/z* 175.0240) (Figure 2). Incubation of 4-hydroxybenzoic acid (M2) with human liver S9 fractions or intestinal microsomes in the presence of UDPGA yielded M3, which was identified as 4-hydroxybenzoic acid glucuronide by comparison with the mass, retention time, and product ions of the authentic standard. 

M4 exhibited an [M−H]^−^ ion at *m/z* 657.1674, that is, 176 amu higher than the [M−H]^−^ ion of catalposide, reflecting catalposide glucuronidation. M4 yielded characteristic product ions at *m/z* 481.1353 (reflecting loss of the glucuronosyl moiety from the [M−H]^−^ ion), *m/z* 319.0823 (loss of glucose from *m/z* 481.1353), *m/z* 205.0499, *m/z* 175.0240, *m/z* 113.0238, and *m/z* 85.0283 (Figure 2). Thus, M4 was identified as catalposide glucuronide. 

**Figure 3:**
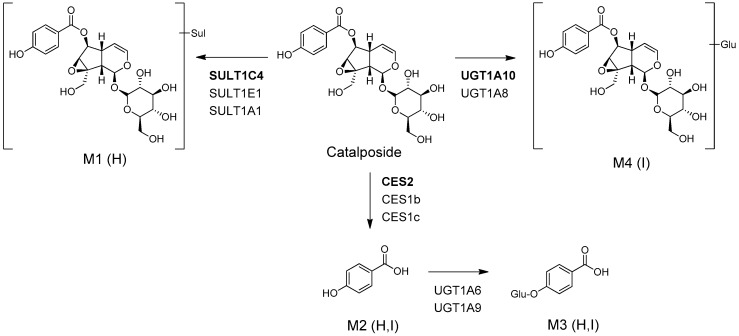
Possible in vitro metabolic pathways of catalposide incubated with human hepatocytes and intestinal microsomes. H: human hepatocytes; I: human intestinal microsomes; Sul: sulfate; Glu: glucuronic acid; CES: carboxylesterase; UGT: UDP-glucuronosyltransferase; SULT: sulfotransferase.

The possible in vitro metabolic pathways of catalposide in humans are shown in Figure 3. Catalposide is metabolized to catalposide sulfate (M1), catalposide glucuronide (M4), and 4-hydroxybenzoic acid (M2); the latter is then further metabolized to 4-hydroxybenzoic acid glucuronide (M3).

### 3.2. Characterization of Human SULT, UGT, and CES Enzymes Involved in Catalposide Metabolism

**Figure 4:**
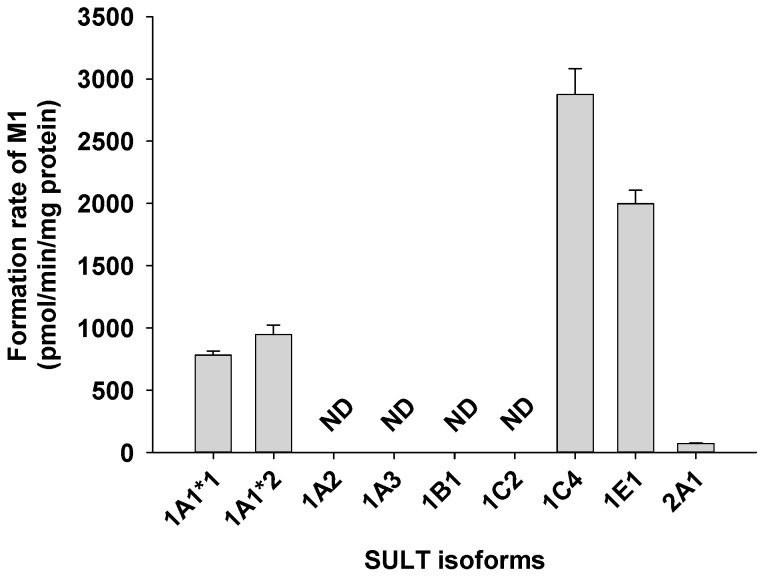
Rate of formation of catalposide sulfate (M1) from 150 μM catalposide by human cDNA-expressed SULT enzymes. All data are means ± SD (*n* = 3). ND: <32 pmol/min/mg protein.

A screen using human cDNA-expressed SULTs 1A1*1, 1A1*2, 1A2, 1A3, 1B1, 1C2, 1C4, 1E1, and 2A1 to assess the formation of catalposide sulfate (M1) from catalposide identified possible roles for SULTs 1A1*1, 1A1*2, 1C4, and 1E1 (Figure 4). 

**Figure 5:**
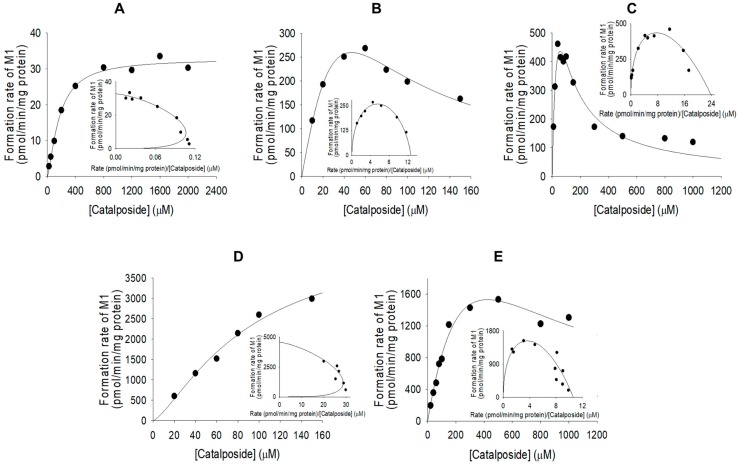
Michaelis–Menten plots of the sulfation of catalposide to catalposide sulfate (M1) by pooled human liver S9 fractions (**A**) and human cDNA-expressed SULT1A1*1 (**B**), SULT1A1*2 (**C**), SULT1C4 (**D**), and SULT1E1 (**E**). Insets: Eadie–Hofstee plots. Each data point represents the average of two determinations.

The formation of catalposide sulfate from catalposide catalyzed by SULTs 1A1*1, 1A1*2, and 1E1 exhibited substrate inhibition kinetics, but the activities of SULT1C4 and pooled human liver S9 fractions fitted the Hill equation (Figure 5). The enzyme kinetic parameters for the formation of catalposide sulfate from catalposide are listed in Table 2. SULT1C4 exhibited a higher affinity for catalposide and more rapid sulfation than did SULT1A1*1, SULT1A1*2, and SULT1E1.

**Figure 6:**
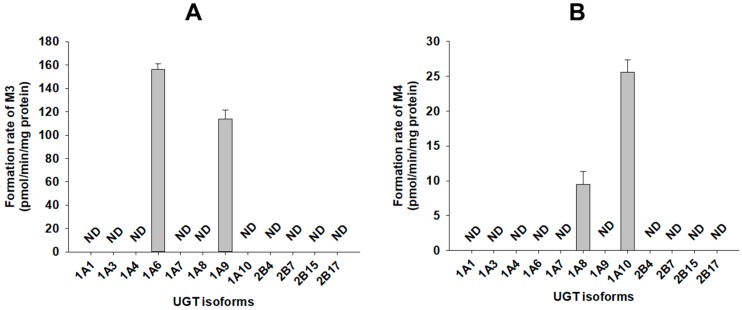
Rates of formation of (**A**) 4-hydroxybenzoic acid glucuronide (M3) from 500 μM 4-hydroxybenzoic acid (M2) and (**B**) catalposide glucuronide (M4) from 400 μM catalposide by human cDNA-expressed UGT enzymes. All data are means ± SD (*n* = 3). ND: <27 pmol/min/mg protein for M3, <0.83 pmol/min/mg protein for M4.

A screen using human cDNA-expressed UGTs 1A1, 1A3, 1A4, 1A6, 1A7, 1A8, 1A9, 1A10, 2B4, 2B7, 2B15, and 2B17 supersomes for the metabolism of 4-hydroxybenzoic acid (M2) to 4-hydroxybenzoic acid glucuronide (M3) identified possible roles of UGT1A6 and UGT1A9 (Figure 6A). The results show that 4-hydroxybenzoic acid glucuronide (M3) was produced on incubation of catalposide with human hepatocytes, liver S9 fractions, and intestinal microsomes. 

A screen using twelve human cDNA-expressed UGT supersomes, to assess the metabolism of catalposide to catalposide glucuronide (M4), identified possible roles of gastrointestinal tract-specific UGT1A8 and UGT1A10 (Figure 6B). The results show that catalposide glucuronide (M4) was produced after incubation of catalposide with pooled human intestinal microsomes, but not human liver S9 fractions. 

**Figure 7:**
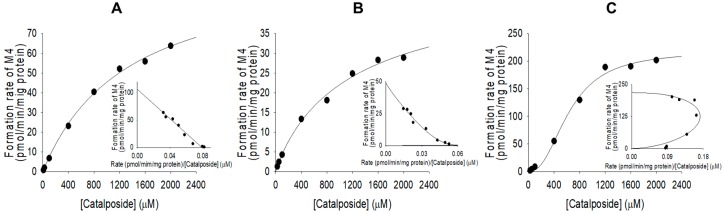
Michaelis–Menten plots for glucuronidation of catalposide to catalposide glucuronide (M4) by pooled human intestinal microsomes (**A**) and human cDNA-expressed UGT1A8 (**B**) and UGT1A10 (**C**). Insets: Eadie–Hofstee plots. Each data point represents the average of two determinations.

Formation of catalposide glucuronide (M4) from catalposide by pooled human intestinal microsomes followed single enzyme kinetics; formation via UGT1A8 and UGT1A10 exhibited Hill equation kinetics (Figure 7, Table 2). 

**Figure 8:**
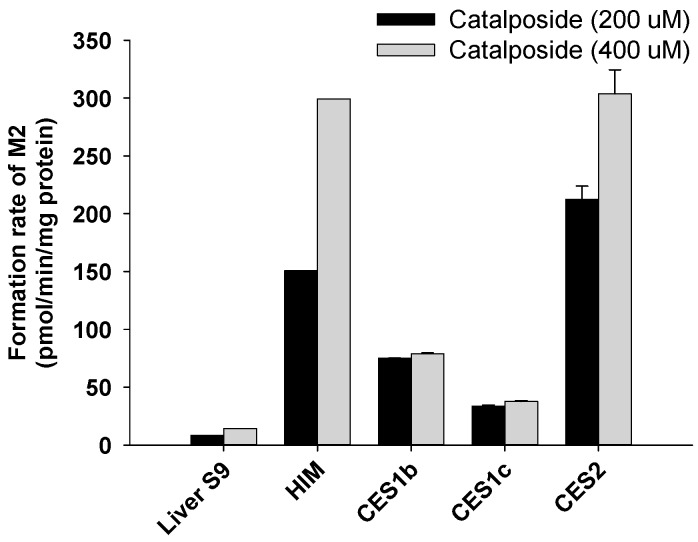
Rates of formation of 4-hydroxybenzoic acid (M2) from 200 μM and 400 μM catalposide in human liver S9 fractions (Liver S9); human intestinal microsomes (HIM); and human cDNA-expressed CES1b, CES1c, and CES2 enzymes. All data are means ± SD (*n* = 3).

4-Hydroxybenzoic acid (M2) was formed from catalposide by pooled human liver S9 fractions; intestinal microsomes; and the CES1b, CES1c, and CES2 enzymes (Figure 8). The rate of formation of 4-hydroxybenzoic acid (M2) after incubation of catalposide with pooled human intestinal microsomes was higher than that after incubation with pooled human liver S9 fractions (Figure 8).

## 4. Discussion

Catalposide was metabolized to catalposide sulfate (M1), catalposide glucuronide (M4), 4-hydroxybenzoic acid (M2), and M2 glucuronide (M3) by human hepatocytes or intestinal microsomes (Figure 3). On the basis of the kinetics of catalposide sulfate (M1) formation from catalposide catalyzed by human cDNA-expressed SULTs 1A1*1, 1A1*2, 1C4, and 1E1 (Figure 4, Table 2), we suggest that SULT1A1, SULT1C4, and SULT1E1 may play major roles in this metabolism. SULT1C4 exhibited higher activity (Cl_int_, 51.0 μL/min/mg protein) in terms of catalposide sulfation than did SULT1A1*1, SULT1A1*2, or SULT1E1 (Cl_int_, 10.6~23.8 μL/min/mg protein) (Table 2). SULT1C4 is highly expressed in the fetal lung and kidney, and at lower levels in the fetal heart, adult kidney, ovary, brain, and spinal cord [21,22,23]. SULT1A1 is the major hepatic SULT (53% of total hepatic SULTs), but is also present in substantial quantities in the small intestine (19% of total SULTs) [23,24]. SULT1E1 is expressed at relatively low levels in the liver (6% of total SULTs) and small intestine (8% of total SULTs), but is the most abundant enzyme in the lung (40% of total SULTs) [23,24]. Catalposide was metabolized to M1 by cytosolic SULTs of both hepatic and extrahepatic tissues. There may be inter-individual variability in catalposide sulfation, given that SULT1A1, SULT1C4, and SULT1E1 polymorphisms are known in humans [25]. SULT1A1, SULT1C4, and SULT1E1 are induced or inhibited by various drugs and chemicals [26,27]; therefore, co-administration of drugs that inhibit or induce expression of these enzymes may affect catalposide sulfation and thus catalposide pharmacokinetics.

Metabolism of catalposide to catalposide glucuronide (M4) was mediated by human cDNA-expressed UGT1A8 and UGT1A10 enzymes, which are confined to the gastrointestinal tract [28,29,30,31,32] (Figure 7), indicating that catalposide glucuronidation was gastrointestinal tract-specific; glucuronidation was not detected after incubation of catalposide with human hepatocytes and liver S9 fractions. UGT1A10-catalyzed catalposide glucuronidation was more extensive (Cl_int_, 0.3399 μL/min/mg protein) than UGT1A8-catalyzed glucuronidation (Cl_int_, 0.0396 μL/min/mg protein) (Table 2). UGT1A10 is more abundant than UGT1A8 in the small intestine (17.3% vs. 0.8% of total UGT protein) and colon (27.4% vs. 1.5% of total UGT protein) [32]. Thus, UGT1A10 may play the major role in glucuronidation of catalposide and UGT1A8 only a minor role. The human UGT1A8 and UGT1A10 enzymes are inhibited by various drugs [30,32,33,34,35]. Therefore, co-administration of drugs that inhibit or induce UGT1A8 and UGT1A10 may affect catalposide glucuronidation. 

CES2, the predominant CES of the intestine, was more active in terms of hydrolysis of catalposide to 4-hydroxybenzoic acid (M2) than were the hepato-predominant CES1b and CES1c enzymes [32,36]. Thus, the rate of formation of 4-hydroxybenzoic acid (M2) was higher when catalposide was incubated with pooled human intestinal microsomes than with pooled human liver S9 fractions.

UGT1A6 and UGT1A9 play major roles in the formation of 4-hydroxybenzoic acid glucuronide (M3) from 4-hydroxybenzoic acid (Figure 6A). Abbas et al. [37] found that UGT1A9 played the major role in metabolism of 4-hydroxybenzoic acid to 4-hydroxybenzoic acid glucuronide. UGT1A6 and UGT1A9 are major enzymes of both the liver and intestine; therefore, 4-hydroxybenzoic acid glucuronide (M3) was identified after incubation of catalposide with either human hepatocytes or intestinal microsomes.

## 5. Conclusions

Catalposide was metabolized to catalposide sulfate (M1), 4-hydroxybenzoic acid (M2), M2 glucuronide (M3), and catalposide glucuronide (M4) via sulfation, glucuronidation, and hydrolysis, on incubation with human hepatocytes, liver S9 fractions, or intestinal microsomes. SULT1A1, SULT1C4, and SULT1E1 formed catalposide sulfate (M1) from catalposide. Gastrointestine-specific UGT1A8 and UGT1A10 played major roles in formation of catalposide glucuronide (M4). CES2 and CES1 catalyzed hydrolysis of catalposide to 4-hydroxybenzoic acid (M2), which was further metabolized to M2 glucuronide (M3) by UGT1A6 and UGT1A9. These results suggest that SULT1A1, SULT1C4, SULT1E1, UGT1A8, UGT1A10, CES2, and CES1 enzymes may play important roles in the pharmacokinetics and drug–drug interaction of catalposide in humans. The pharmacokinetics of catalposide may be dramatically affected by the co-administration of inhibitors or inducers of UGTs, CESs, or SULTs.

## Figures and Tables

**Table 1:** Molecular formulae, deprotonated molecular ions ([M−H]^−^), mass errors, retention times (t_R_), and product ions of catalposide and its metabolites were identified after incubation of catalposide with human hepatocytes and intestinal microsomes.

Metabolite	Formula	Exact Mass [M−H]^−^ (*m/z*)	Error (ppm)	t_R_ (min)	Product Ions (*m/z*)
Catalposide	C_22_H_26_O_12_	481.1349	−0.6	10.26	319.0822, 205.0497, 137.0239
M1	C_22_H_26_SO_15_	561.0921	0.2	8.74	481.1349, 319.0822, 205.0499, 137.0239
M2	C_7_H_6_O_3_	137.0239	−3.6	4.07	93.0339
M3	C_13_H_14_O_9_	313.0569	1.3	2.02	193.0345, 175.0240, 137.0239, 113.0239, 85.0283
M4	C_28_H_34_O_18_	657.1674	0.3	6.41	481.1353, 319.0823, 205.0499, 175.0240, 137.0239, 113. 0238, 85.0283

**Table 2:** Kinetic parameters for the formation of catalposide sulfate (M1) and catalposide glucuronide (M4) from catalposide in pooled human liver S9 fractions, intestinal microsomes, and human cDNA-expressed sulfotransferase (SULT) or UDP-glucuronosyltransferase (UGT) enzymes.

Enzyme	*K*_m_ (μM)	*V*_max_	*K*_i_ (μM)	*n*	*Cl*_int_
Sulfation of catalposide to M1					
SULT1A1*1	162.0	2046.8	13.7	-	12.6
SULT1A1*2	50.5	1203.2	65.5	-	23.8
SULT1C4	89.8	4576.2	-	1.4	51.0
SULT1E1	456.6	4840.0	391.4	-	10.6
Human liver S9 fractions	169.7	32.8	-	1.4	0.19
Glucuronidation of catalposide to M4					
UGT1A8	1230.1	48.7	-	0.9221	0.0396
UGT1A10	641.7	218.1	-	2.3	0.3399
Human intestinal microsomes	1341.3	106.2	-	-	0.0792

*V*_max_: pmol/min/mg protein; *Cl*_int_: μL/min/mg protein; *n*: Hill coefficient.
